# Primary pulmonary artery tumors easily misdiagnosed as pulmonary embolism: A review

**DOI:** 10.1097/MD.0000000000033337

**Published:** 2022-04-07

**Authors:** Xiuqing Liu, Xuhan Liu, Ruirui Li, Weihua Zhang

**Affiliations:** a Department of Cardiovascular Medicine, The First Hospital of Jilin University, Changchun, China.

**Keywords:** imagine diagnosis, pathological diagnosis, primary pulmonary artery tumor, pulmonary artery sarcoma, pulmonary embolism, surgical treatment

## Abstract

Primary pulmonary artery tumors (PPATs), originating from the pulmonary artery intima, are rare tumors characterized by pulmonary artery luminal occlusion and pulmonary hypertension. Diagnosis of this rare entity is a challenging dilemma with the need for a high expertise in the radiological and pathological identification of PPATs. computed tomographic pulmonary angiography of PPATs may show filling defects, which are easily misdiagnosed. The radionuclide scan, along with other imaging examinations, can assist with the diagnosis, but the pathological diagnosis requires a puncture or surgical resection. Most primary pulmonary artery tumors are malignant, with poor prognosis and lack of specificity in clinical manifestations. However, there is no unified understanding and standard for diagnosis and treatment. In this review, we discuss the status, diagnosis, and treatment of primary pulmonary artery tumors, as well as how clinicians can better understand and treat the disease.

## 1. Introduction

Pulmonary embolism (PE) is the most common filling defect seen on computed tomographic pulmonary angiography (CTPA). Primary pulmonary artery tumors (PPATs) can mimic PE on imaging and clinical presentation. PA tumors, especially malignant ones, have radically different treatments and a more severe prognosis from PE. PPATs are a rare disorder of undetermined etiology, originating from the main pulmonary artery or the tissue of the pulmonary valve. The first case was reported by Mandelstamm through autopsy in 1923.^[1]^ So far, only a few hundred cases of such diseases have been reported in the literature, and most of them are individual cases, and the vast majority are malignant tumors. Pulmonary artery sarcoma (PAS) is the most common in kinds of literature, accounting for <400 cases.^[2]^ Other types such as primary pulmonary artery myxoma, Primary papillary fibroma, and Primary pulmonary artery chorionic carcinoma have only been rarely reported.^[3]^ The main imaging manifestations are irregular soft tissue masses in the lumen, which is similar to PE. As the most common primary pulmonary artery sarcoma (PPAS), the prevalence rate reported in the literature is only 0.001% to 0.030%,^[4]^ considering that many cases are often misdiagnosed as PE in the preoperative examination, or there are several cases without pathological examination, and other types of PPATs are mostly sporadic cases. Therefore, the actual prevalence rate of PPATs is still unclear. The average age of onset of PPATs is 48 years old (range 13–86 years old).^[5]^ Some studies have shown that there is no significant difference between male and female patients, and Liu MX et al^[6]^ also reported that most of the patients are female (2:1). The vast majority of cases occurred in adults and a few in children.^[3]^ The prognosis of this kind of disease is poor, and the clinical diagnosis can only be confirmed by puncture or surgical resection of biopsy tissue for pathological examination, so its early diagnosis is extremely difficult.^[5]^ With the increase in the prevalence rate of PE and the improvement of clinicians awareness of it, PPATs are usually misdiagnosed as PE because of their similarity in imaging and clinical manifestations with PE.^[7,8]^ That may lead to inappropriate treatments for patients, such as prolonging anticoagulation time or delaying surgical treatment, which may delay the illness.^[9]^ However, there is no unified understanding and standard on the clinical features, diagnosis, and treatment of PPATs. In this review, we present clinical, imaging, and histopathologic features of benign and malignant PPATs, emphasizing differentiating features from PE. We also describe available diagnostic and treatment methods for PPATs. So we hope to help clinicians better understand the disease through the relevant literature.

### 1.1. Clinical characteristics

Han Y et al^[10]^ thought PPATs often occur in the main pulmonary artery and the pulmonary valve, which can extend to the bifurcation of the pulmonary artery and the left and right pulmonary trunks. A few can retrograde spread to the right ventricular outflow tract through the pulmonary artery lumen, or originate from the right ventricular outflow tract, but they are extremely rare. Histologically, it can be divided into benign and malignant tumors, especially malignant tumors. There are only a few primary benign tumors of the pulmonary artery, including pulmonary artery angiomyxoma, pulmonary artery myxoma, pulmonary artery lipoma, etc.^[7]^ PPAS is the most common of primary pulmonary artery malignancies, but because primary pulmonary artery malignancies are extremely rare, primary pulmonary artery malignancies are limited to sarcomas.^[7]^ It has been suggested that PPAS may originate from mesenchymal cells in the arterial intima, and have the potential for multi-directional differentiation. Therefore, PPAS contains many different pathological types.^[10]^ Among them, undifferentiated sarcoma was the most common type, accounting for 34% of all PAS, followed by fibrosarcoma (21%), leiomyosarcoma (20%), rhabdomyosarcoma (6%), mesenchymal histiocytoma (6%), chondrosarcoma (4%), angiosarcoma (4%), osteosarcoma (3%), and malignant fibrous histiocytoma (2%), etc.^[7]^ However, these tumors have common clinical characteristics, and an overall poor prognosis.

Clinical manifestations of patients with PPATs are not specific, and the differences in clinical symptoms may depend on the size and anatomical location of the tumors. Patients with PPATs usually present with common cardiovascular or pulmonary symptoms, such as dyspnea, cough, chest pain, and hemoptysis. Dyspnea is the most common symptom. Cough, chest pain, hemoptysis, and syncope are also common,^[8]^ symptoms of hemoptysis may suggest that the tumor invades the adjacent bronchi. Palpitation is the main symptom in some patients.^[11]^ Some patients have non-pulmonary systemic symptoms of fever and cachexia or symptoms caused by local spread, such as tachyarrhythmia, conduction defects, pericardial effusion,^[12]^ inflammatory anemia,^[13]^ and clubbed-fingers. A few patients have no obvious symptoms in the early stage, when there is almost complete occlusion near the pulmonary artery valve and tumor growth persists, symptoms may suddenly appear, possibly manifesting as severe hypotension or shock.^[8]^ Given the similar clinical manifestations to PE, PPATs may be diagnosed as acute PE. It has been reported that approximately 50% of patients with PPATs were initially diagnosed with acute PE^[8]^ and received anticoagulation therapy. When anticoagulant therapy fails to improve the patient’s condition, it may lead to further examination. It is possible that “the thrombus” continues to grow based on adequate anticoagulation. So that PPATs are finally diagnosed after inappropriate treatment for a long time. There is currently no literature indicating whether anticoagulation affects clinical course. As with all other cancers, symptoms of local infiltration and distant metastasis may exist and vary with the scope of the disease and anatomic location,^[8]^ the lung is the most commonly affected area, most likely caused by thrombus,^[3]^ regional lymph node, liver, pancreas, brain, and kidney metastases have also been reported. There may be no obvious positive signs in the patient’s physical examination. However, when the treatment is delayed due to misdiagnosis and pulmonary hypertension (PH) and right ventricular dysfunction are combined, there may appear signs as a systolic murmur in the pulmonary valve area, cyanosis, jugular vein flaring, hepatomegaly, and edema in both lower limbs.^[14]^ In contrast, clinical manifestations of primary pulmonary artery benign tumors have no obvious specificity, and symptoms of dyspnea, chronic recurrent cough, and hemoptysis have been reported,^[3]^ which are not obviously different from symptoms of malignant tumors in general. Routine laboratory tests such as D-dimer, erythrocyte sedimentation rate, C-reactive protein, and tumor markers did not show good diagnostic value, some showed slightly elevated neuron-specific enolase (less than twice the upper limit).^[11]^ Electrocardiogram can usually indicate nonspecific manifestations such as right ventricular pressure elevation, right ventricular hypertrophy, and changes in the ST segment and T wave. Therefore, it is difficult for clinicians to suspect the diagnosis of PPATs based on the above-mentioned clinical manifestations and routine laboratory tests.

### 1.2. Diagnosis

**Figure 1.**
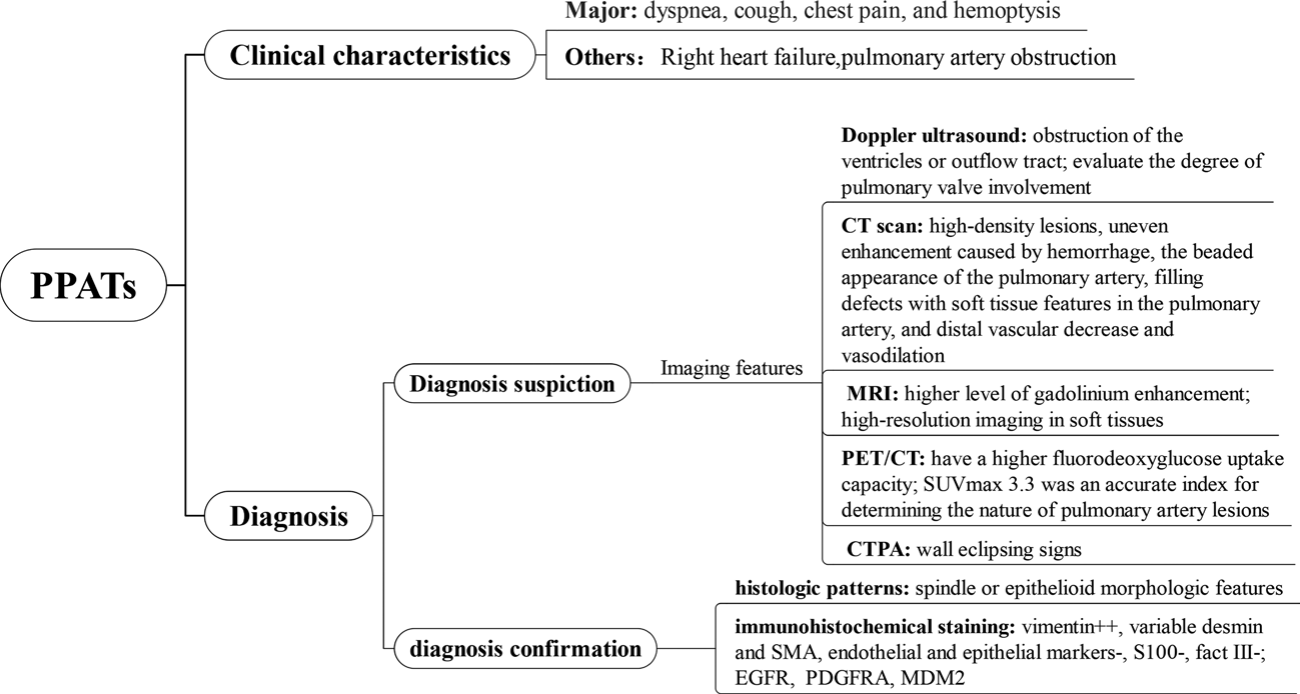
The procedure for diagnosis and management of primary pulmonary artery tumors.

As a rare disease with a low incidence, PPATs can only be definitively diagnosed by biopsy or surgical excision. In the absence of specific clinical manifestations and laboratory tests, early diagnosis is extremely difficult. Patients with early symptoms associated with increased pulmonary vascular resistance tend to be diagnosed with common diseases. Meanwhile, PPATs are often misdiagnosed as acute or chronic PE in pulmonary augmentation CT with lung filling defects. Therefore, we should strengthen the understanding of relevant imaging examinations, such as echocardiography, CTPA, magnetic resonance imaging (MRI), and positron emission tomography/computed tomography (PET/CT), and also the understanding of immunopathology, to enhance the understanding of the disease and reduce the misdiagnosis rate (Fig. 1).

## 2. Imaging features

### 2.1. Doppler ultrasound

Cardiac echocardiography is essential in the diagnosis and evaluation of patients with PPATs and is used to detect obstruction of the ventricles or outflow tract,^[5]^ which can show right ventricular dilatation and obstruction of the outflow tract or pulmonary artery trunk. However, thrombus and tumor tissues in pulmonary trunk could not be accurately distinguished. Cardiac echocardiography is the most common examination in clinical, and being familiar with the operation and diagnostic significance of this examination can provide more clinical ideas for clinicians. Transesophageal echocardiography can also be used for the diagnosis of PPATs, clarify the physiological significance of the lesions, and evaluate the ventricular and cardiac valve functions.^[15]^ In addition, the application of intravascular ultrasound is helpful to evaluate the degree of pulmonary valve involvement or to distinguish between interlayer, mural thrombus, or malignant tumors of the vascular.^[16]^ On the basis of familiar with echocardiography, it may be possible to better assess disease involvement and severity in conjunction with other examinations.

### 2.2. Computed tomography scan (CT scan)

Contrast-enhanced CT scan of the chest is usually the first imaging technique to be performed in patients with respiratory symptoms, CT scan findings support the diagnosis of PPATs rather than PE, including aspects of both morphology and density. On the plain scan, the pulmonary artery showed iso-low-density filling defects (average 30HU, which is difficult to distinguish from the image of thrombosis).^[15]^ Patchy and delayed enhancement with more pronounced delayed enhancement in the venous phase (an average increase of 25HU) was observed on CT angiography, which suggested necrosis, hemorrhage, or ossification.^[15]^ It was found that a large number of patients with PPAS found filling defects occupying the entire lumen diameter of the main or proximal pulmonary artery on CT images, which is rare in patients with PE.^[5,7,17]^ In this case, CT images may be irregularly distributed or with high-density filling defects, and the images may show lobulated or heterogeneous hilar masses starting from the pulmonary artery trunk or main pulmonary artery and extending to the peripheral arteries.^[15]^ The tumor vascularity, hemorrhage, necrosis, and ossification in pulmonary artery sarcoma on CT scan show that heterogeneous enhancement is helpful for us to distinguish pulmonary artery tumors from PE disease. For benign tumors of the pulmonary artery, such as angiomyxoma, the results of the CT scan depend on the origin of the lesion. Tumors of extraluminal origin may present as nodules and invasive masses, while intraluminal growth may show intravascular filling defects.^[18]^ In conclusion, the differential manifestations of pulmonary artery tumors distinguished from PE by CT scan include high-density lesions, uneven enhancement caused by hemorrhage, the beaded appearance of the pulmonary artery, filling defects with soft tissue features in the pulmonary artery, and distal vascular decrease and vasodilation. In addition to contributing to the diagnosis of primary pulmonary artery tumors, CT scan can detect the local spread of the tumors to the mediastinum and parenchyma and evaluate cardiac function in patients with PPATs by indirect imaging features of right ventricular pressure overload, such as right ventricular dilatation and contrast agent regurgitation.^[5]^ Clinicians should be familiar with and master the CT imaging features differences of pulmonary embolism disease and pulmonary artery tumors to quickly grasp the imaging features for differential diagnosis and avoid misdiagnosis.

### 2.3. MRI

With the popularity of advanced cardiac imaging technology, despite primary pulmonary artery tumors being very rare, MRI, as an advanced imaging modality, can timely and accurately diagnose or reduce the types of diseases that need to be identified.^[19]^ Compared with thrombus, PPATs have a higher level of gadolinium enhancement on MRI images, and gadolinium enhancement of MRI helps to identify intraluminal involvement of the PPATs. This enhancement may be related to tumor differentiation and mucus matrix content, and post-gadolinium inversion recovery magnitude and phase-sensitive images showing heterogenous hyperenhancement of the mass consistent with malignant tumors.^[20]^ In the 2017 literature, Liu et al^[21]^ described the specific manifestation of pulmonary artery tumors on MRI, that is, the grape-shaped appearance of the pulmonary artery, which was the result of the extension of the intraluminal filling defects to the peripheral arteries, and found the signs of cardiac invasion, which had strong specificity for the diagnosis of PAS. The morphology of PPAT is usually dilated growth with corresponding pulmonary artery widening, which helps to distinguish PPAT from PE. In addition, the proximal part of pulmonary artery tumor lesions is often convex and lobulated, and the distal part is aneurysmal expansion or grape-like, suggesting that these changes may be caused by local accumulation, expansion and infiltration of tumor tissues.^[6]^ Soft tissue nodules or masses within the pulmonary arteries, as well as the relationship between the diseased tissue and the pulmonary valve or right ventricle, could be clearly visualized on MRI from the main pulmonary trunk to both pulmonary arteries. At the same time, it also provides opportunities to observe the tumor activity through blood flow. Since MRI enables high-resolution imaging in soft tissues, it is more advantageous when distinguished from PE,^[15]^ and can be used for posttreatment follow up and monitoring of patients with primary pulmonary artery tumors to assess residual or recurrent tumors.^[15,22]^ Although MRI has a good ability to describe organizational features, it still has some shortcomings, including lower spatial resolution and longer breath-holding time. If we consider that most patients examined for this condition have severe dyspnea, this examination may not be chosen.^[15,23]^ In short, with the popularization and application of magnetic resonance imaging technology, we should make full use of the specific manifestations of soft tissues in MRI, to help us to make the differential diagnosis of rare diseases and deepen our understanding of the diseases.

### 2.4. PET/CT

PET/CT is often used to confirm the suspicion of PE and to differentiate PATs from PE based on the uptake of radiopharmaceuticals by tumors. Pulmonary artery malignancies are more likely to be supplied by a large number of vessels and have higher metabolic activity than pulmonary embolic diseases; therefore, PET-CT, as a metabolic imaging means, can determine the degree of malignancy of the tumors.^[24]^ PET/CT imaging can show strong uptake of fluorodeoxyglucose in the lesion site and can also show metastatic focus. Although acute PE may also have some fluorodeoxyglucose uptake capacity, pulmonary artery malignant tumors have a higher fluorodeoxyglucose uptake capacity.^[25]^ Some experts have proposed that the SUVmax 3.5 is the critical point, and the sensitivity, specificity, and accuracy for identifying PPATs and PE are 100%.^[26]^ The study, which used 18F-fluorodeoxyglucose (FDG) PET/CT for comprehensive analysis for the first time to distinguish malignant PPATs from PE, showed that SUVmax was an accurate index for determining the nature of pulmonary artery lesions when the critical value of SUXmax was 3.3.^[26]^ Xi et al^[24]^ suggested that the sensitivity of PET/CT could reach 98.4%, but it is noteworthy that a false-negative case (SUVmax2.6) was reported in the literature.^[27]^ In addition, several other literatures have also stated that PAS has a poor ability to uptake FDG, which may be due to its low cell density and rich mucus-like tissue.^[16,28,29]^ Therefore, the actual sensitivity may be lower than 98.4%. Except for PE, benign lesions of the pulmonary artery include other rare diseases that may present with varying degrees of FDG uptake, for example, a case of suppurative granuloma with a high uptake rate (SUVmax 4.1) was reported in the literature.^[30]^ Few reports suggest that benign pulmonary artery lesions have a strong uptake ability of FDG.^[30,31]^ Tumor necrosis, hemorrhage, or calcification may also be associated with rare FDG uptake in tumors, with the 18F-FDG uptake rate being significantly higher in some patients than that in patients with thrombosis. The exact location of the tumors is important for complete resection of the PATs, it can help to determine the survival range of the tumors, thus optimizing the surgical plan. It often uses PET/CT for staging in other tumors, and it has been reported in the literature that PET/CT shows local recurrence of lung metastasis, which also reflects the role of PET/CT in rare tumor restaging.^[32]^ However, the tumor burden near the right ventricle may be confused by the high uptake of FDG in the myocardium, which is limited by the low sensitivity and the potential for false positives compared to MRI,^[33]^ such as benign lipomatous hypertrophy is highly metabolically active.^[34]^ In conclusion, the specificity of 18F-FDG PET/CT imaging in the differential diagnosis of malignant pulmonary artery tumors from PE far exceeds its sensitivity in the existing literature. Thus, it may be necessary to evaluate the cardiac influences of the tumor in combination with other examination methods such as MRI.

### 2.5. CTPA

CTPA is currently considered to be a useful tool for differentiating PPATs from pulmonary thromboembolic diseases. Although both show filling defects of the pulmonary artery on imaging, PPATs can extend to the main pulmonary artery trunk and the right ventricular outflow tract and tend to occupy the entire lumen, with localized tumor-like dilatation, widened pulmonary artery diameter, and the possibility of forming a characteristic acute angle with the vessel wall, and tumor proximal ends usually have lobulated, raised margins, while pulmonary artery thrombosis usually has straight cup-shaped edges^[6,8,25,35,36]^ and an obtuse angle with the vessel wall.^[37]^ In addition, approximately 90% of pulmonary artery neoplastic lesions involve more than 2 parts of the pulmonary artery, most commonly affecting the lobar pulmonary artery (85%), followed by the right pulmonary artery (71%), the left pulmonary artery (65%), and the right ventricular outflow tract (10%).^[6]^ In 1 study, CTPA was performed in 12 patients with PPAS, 156 patients with chronic pulmonary thromboembolism combined with pulmonary hypertension, and 426 patients with acute PE, the results showed that all patients with PPAS had “wall eclipsing signs,” while none of the patients with acute and chronic thromboembolism showed this sign.^[38]^ This sign is defined as the presence of the following 3 findings: a low-density intraluminal mass of the pulmonary trunk, left pulmonary artery, or right pulmonary artery with near complete occlusion; proximal protrusion of the mass toward the right ventricular outflow tract; and eclipsing of 1 or both walls of the involved artery before the lesion infiltrates beyond the artery.^[35,39]^ Therefore, the “wall eclipsing sign” on CTPA is considered to be the specific manifestation of pulmonary artery tumors.^[35,38–40]^ With this manifestation, it is possible to accurately differentiate PPATs from PE, which requires that both Radiologists and clinicians be familiar with the characteristics of this disease on CTPA, to improve the accuracy of the diagnosis.

## 3. Pathology of tissue biopsy

As mentioned above, imaging may only indicate the possibility of diagnosis and help to determine the extent of involvement, and histopathological and immunohistochemical findings are needed to determine the final diagnosis.^[37]^ Most of the tissues were obtained by surgery or autopsy.^[3,41]^ Given the risk of potential complications during the biopsy process, such as CT scan-guided transthoracic aspiration and transbronchial biopsy, the role of a preoperative biopsy in patients with pulmonary artery tumors is controversial. Some studies have shown that endobronchial ultrasound-guided transbronchial needle aspiration (EBUS-TBNA) can obtain a pathological diagnosis. EBUS-TBNA allows better visualization of the tumor and vascular flow via doppler ultrasonography,^[42]^ and the obtained cytological materials can be quickly and in-site evaluated to facilitate accurate and early diagnosis of PATs,^[9,43]^ which was speculated to be a minimally invasive and feasible method for assessing intravascular lesions.^[44,45]^ However, it has also been stated that PH caused by occlusive lesions increases the risk of bleeding complications.^[42,46,47]^ Thus, in the diagnosis of PPATs, the limited sample sizes make the relevant literature less reported, and there is still controversy about the application of this method in the diagnosis of this disease.^[42]^ Another technique using the endovascular catheter-guided forceps has been able to successfully detect intimal sarcomas of the pulmonary artery in more than 75% of the patients,^[41,48]^ which is an effective and safe diagnostic tool for patients with PPATs. Since the tumor tissue of pulmonary artery sarcoma is always covered with necrotic tissue or in situ thrombus, it can be repeated to obtain the actual lesion site by the intravascular catheter biopsy, intravascular catheter biopsy should be performed for early diagnosis when PPATs are suspected.^[48,49]^ The endovascular catheter-guided forceps biopsies require extensive training in pulmonary arteries catheterization, as well as the use of biopsy devices with considerable catheter sizes to extract sufficient tumor tissues.^[50]^ It may be an interesting method for patients with inoperable or suspected disease recurrence.^[5,51]^ Due to the rarity of PPATs, there is no clear consensus on the diagnosis of PPATs. However, with the advancement of medical technology, various methods for obtaining tissues are constantly being tried, and there are still disputes on safety and reliability, which requires continuous technical training, and accurate estimation and processing ability for possible risks. So that it can be more skilled and safer in the diagnosis process.

The histologic patterns of PPAS are heterogeneous. These can show heterologous elements like osteosarcoma or chondrosarcoma. In the most common, undifferentiated form, tumor cells are identified as exhibiting spindle or epithelioid morphologic features cytologic atypia, and pleomorphism. When compared with an organizing thrombus, the thrombus usually contains fibrin, RBCs, and lines of Zahn, as well as endothelial cell growth.^[7,25]^ The immunohistochemical staining for PAS revealed that vimentin was strongly expressed in all patients with PAS,^[5,52]^ with positive expression of desmin and smooth muscle actin (SMA) in varying degrees, and negative expression of endothelial and epithelial markers as well as S100 protein.^[13,52]^ In addition, murine double minute 2 (MDM2) is a proto-oncogene that is a negative regulator of the p53 protein. Immunohistochemical analysis showed that MDM2 was expressed in the most PASs and related to the amplification of the MDM2 gene in 12q13-14, indicating that the MDM2/p53 pathway might be a mechanism for the pathogenesis of PAS.^[5,52]^ The fluorescent in situ hybridization (FISH) analysis is a reliable and sensitive technique for detecting MDM2 amplification.^[53]^ Using FISH technology in patients with pulmonary artery sarcoma, the positive rate of platelet-derived growth factor receptor α (PDGFRA) amplification was 81%, the positive rate of MDM2 amplification was 65%, and the positive rate of the epidermal growth factor receptor (EGFR) amplification was 76%. Noteworthy, the activation of EGFR and PDGFRA usually coexists with the overexpression of MDM2, which can improve the accuracy of the diagnosis of PAS.^[53]^ A literature study performed the cytopathological examination of tissues obtained through EBUS-TBNA, which confirmed that tumor cells were positive for vimentin, desmin, and SMA, and chromosome in situ hybridization on the cells showed significant amplification of MDM2 gene locus, which was consistent with the above view, thus confirming the diagnosis of PAS.^[9]^ In a case of a primary malignant solitary fibroma, the immunohistochemical staining showed positivity for vimentin, CD34 antigen, and CD99 antigen of tumor tissue.^[37]^ It has also been reported in the literature that RUNX-1, nestin, WT1, and CD44 were expressed in the PAS.^[54]^ The pathological diagnosis of PPATs is based on the tumor’s location, morphology, and immunohistochemistry (vientin+, desmin+/−, factor VIII−, SMA+/−, CD31−/+, etc). Special cytogenetic changes, including MDM2 and PDGFRA amplification in FISH or comparative genomic hybridization analysis, can further confirm the diagnosis.^[5,52]^ With the research on immunohistochemistry, it is believed that more specific markers will be found in PPATs to improve diagnostic accuracy.

## 4. Treatment

### 4.1. Surgical management

Pulmonary artery benign tumors are usually treated with surgery, which can be cured by surgical resection and has a low local recurrence rate.^[7]^ In contrast, the malignant tumors of the pulmonary artery have high malignancy and poor prognosis, with an average survival rate of 17 months after diagnosis.^[55]^ Without surgical intervention, the average survival time after diagnosis is only 1.5 months.^[13,14]^ Due to the lack of clinical trials with sample data for PPATs,^[2]^ there is no clear consensus on the treatment, and the treatment decision depends on the patient’s symptoms, the size of the lesion, its distant spread, and the possibility of the clinical team to determine a successful treatment.^[7]^ The standard treatment is early and aggressive surgery, which aims to complete surgical resection with clear margins.^[13,14]^ Surgical data for PAS have been extrapolated from PATs with varying incidences.^[10,14,56]^ It has been reported that the survival rate of patients with successful surgical resection can be increased to 8 to 36 months.^[57]^ Patients who attempted radical resection had a longer OS than patients with incomplete resection (median OS was 36.5 months vs 11 months).^[13]^ In line with these outcomes, in a case series of the 28 patients with PAS, 18 underwent surgical treatment (14 underwent pulmonary endarterectomy and 4 underwent radical tumor resection), and the median OS tended to favor the surgical approach (20 months vs 9 months).^[5]^ Currently, the reported surgical methods include unilateral pneumonectomy, lobectomy, radical tumor resection combined with pulmonary artery reconstruction, pulmonary endarterectomy, and combined cardiopulmonary transplantation.^[56–60]^ The choice of surgical approach mainly depends on the location and scope of the tumors as well as their metastasis.^[14,56]^ Some experts believe that the survival time of PPAT patient with tumor resection combined with pulmonary endarterectomy is better than that of patients with tumor resection alone.^[60]^ For most patients with PPATs, the tumor cells originate from the proximal pulmonary artery intima and diffuse to the distal end with the intima. Compared with simple tumor resection, pulmonary artery endarterectomy can restore blood flow in the affected area of the lung, ensure adequate oxygenation, and relieve pulmonary artery pressure. As a palliative treatment, it can protect the pulmonary vascular bed and reduce the incidence of pulmonary artery pressure.^[14,61]^ Since patients of PPATs with PH generally have bilateral lesions, multiple medical centers have also emphasized the simultaneous removal of tumors and bilateral pulmonary endarterectomy.^[14,61]^ Even in patients with unilateral lesions without PH, tumor cells may have been planted bilaterally at the time of onset.^[56,62]^ Radical tumor resection and pulmonary artery reconstruction are the most promising treatments for PPATs, which may require the resection of important structures, such as pulmonary valves or the right ventricular outflow tract.^[63]^ Resection of metastatic lesions distal to the implantation site showed a higher survival rate compared with distal embolectomy.^[60]^ Combined cardiopulmonary transplantation is also a treatment, but the success rate is very low. Survival after surgical resection seems to be related to local recurrence rather than progression through metastasis.^[7]^ Long-term survival data of ≥ 7 years after the initial operation have been reported, and systemic metastasis is rare. Therefore, it is recommended for reoperation or resection of metastatic lesions.^[56,64]^ The majority of patients were recommended to use low-molecular-weight heparin for thromboembolism prevention for 6 weeks after the operation.^[56]^

## 5. Chemotherapy therapy

Although surgery can prolong the median survival time of patients, the long-term prognosis is still poor. Mussot et al^[14]^ reported the 3-year survival rate and 5-year survival rate of 31 cases of PAS are only 29% and 22% respectively. Some experts believe that the reason why surgery alone cannot achieve good results is the delayed diagnosis, it is difficult for surgeons to remove it completely with the progress of the tumor. After surgical resection, reducing the tumor load is also very important during treatment, it can be seen that chemotherapy and radiotherapy also seem to be effective treatments.^[11]^ However, there are no standard chemotherapy guidelines. Currently, the chemotherapeutic regimen combining adriamycin and ifosfamide was demonstrated to be effective regardless of the histological subtype of these tumors.^[55]^ The use of other agents, including carboplatin, epirubicin, cyclophosphamide, gemcitabine, dacarbazine, etoposide, and vinorelbine, have also been reported in some types of PPATs with certain effects.^[7,13,65]^ Some experts believe that when some patients are unable to undergo surgery with complete tumor resection, neoadjuvant chemotherapy is recommended to improve the prognosis of patients with locally advanced soft tissue sarcoma and make surgery easier.^[66]^ It has been reported that neoadjuvant chemotherapy has led to operation on patients who were initially inoperable and had a positive effect on survival.^[67]^ Adjuvant therapy for high-risk tumors with a depth or height of more than 5 cm has been recommended in the literature.^[66]^ Some authors emphasized the use of chemotherapy and/or radiotherapy on an individualized basis, while others emphasized that adjuvant therapy was recommended whenever possible to prolong the remission period.^[14,56,60,61]^ Available data confirm that the median survival rate of patients receiving adjuvant therapy is improved than that of patients undergoing surgery only, which indicates that the treatment is effective.^[62,68]^ Many case reports have been published with successful results in favor of chemotherapy in the postoperative setting, especially in patients with residual lesions with doxorubicin-based chemotherapy.^[69,70]^ Delayed chemotherapy for relapse was also demonstrated to be successful in a patient who was treated with amrubicin and ifosfamide.^[71]^ Adriamycin-based chemotherapy remains the most effective strategy for soft tissue sarcoma in the presence of tumor metastasis.^[72]^ The aggressive nature of PPATs and the pulmonary complications associated with surgery have led to the limited treatment of metastatic patients.

## 6. Radiation therapy

Radiotherapy is a necessary part of the treatment for soft tissue sarcoma. For patients with a high-risk of recurrence, radiotherapy is recommended as an adjuvant treatment,^[5]^ and the indications for PPAT still need to be further explored. Secondino et al^[62]^ thought that more than 80% of patients with PPATs received adjuvant therapy, whether 6 or 4 cycles of a chemotherapeutic regimen combining adriamycin and ifosfamide, followed by radiation therapy of 60GY. The median survival time for both groups was 26 months with a range of 10 to 21 months and 4 to 55 months for the chemotherapy alone or concomitant chemoradiotherapy respectively with also a trend towards better survival in patients who received additional postoperative therapy. In addition to its effective role in the postoperative environment, radiotherapy alone can also prolong the survival time in some cases where chemotherapeutic drugs are resistant.^[5]^ Complete regression of tumors after surgery followed by chemoradiotherapy was also reported.^[73]^ In conclusion, multiple safe and effective treatment methods should be integrated as far as possible for patients with postoperative recurrence of PPATs who cannot completely remove the tumor or have a high-risk of postoperative recurrence. However, it is worth noting that surgical treatment remains the preferred option despite the positive effects of chemotherapy and radiation on the patient’s survival rate, chemotherapy and radiotherapy only play a supplementary role, allowing inoperable patients to operate after adjuvant treatment. Therefore, the comprehensive application of multiple treatment regimens may play a positive role in improving the prognosis (Fig. 1).

## 7. Immunotherapy

With the discovery of potentially targetable genetic alterations (MDM2, PDGFRA, and EGFR)^[5,9,51]^ in patients with PPAS, it is considered that the MDM2-p53 pathway is closely related to the occurrence of PPAS,^[52]^ which makes the targeted therapy very attractive in PPATs. In addition, in vitro immunoassays of primary sarcoma tumor cells have shown that both dasatinib and imatinib have strong inhibition effects on tumor cells, most likely due to the inhibition of downstream extracellular signal–regulated kinase 1/2 (ERK1/2) and serine/threonine kinase (AKT) signaling pathways.^[53]^ Van Dievel et al^[74]^ treated 4 patients with metastatic PPAS with PDGFRA mutation with imatinib, of which 1 patient obtained partial remission. Due to the clonal heterogeneity of PPATs and the coexistence of multiple genetic changes (MDM2, PDGFRA, EGFR), targeting multiple tyrosine kinase inhibitors becomes a necessity and an innovative approach in these tumors to improve the oncological outcomes of these dismal tumors.^[5]^ Pazopanib, a multitargeted tyrosine kinase inhibitor including PDGFR, is approved for the second-line therapy of advanced non-adipocytic soft tissue tumors after doxorubicin and ifosfamide failure.^[75]^ A retrospective analysis by Kollar et al^[76]^ of 2 patients with endometrial sarcoma, out of 52 patients with vascular tumors, showed partial remission in both patients in the second-line metastatic disease after receiving pazopanib treatment, but there were no relevant reports of patients with PPATs. Further studies are needed on the efficacy and safety of possible targeted therapeutic agents, and the exploration of more biomarkers for PPATs and therapeutic targets may be the direction in the future.

## 8. Conclusion

PPATs are rare in the clinic and have an extremely poor prognosis, early diagnosis is difficult, and it is easy to be misdiagnosed as PE. Therefore, it is important to consider PPATs in the differential diagnosis of PE to improve the survival rate of patients. When considering that there are persistent vascular filling defects and long clinical durations after adequate anticoagulant therapy, it is recommended to conduct a rapid and comprehensive examination by echocardiography, CT, MRI, and PET/CT as soon as possible in order to improve the early diagnosis rate and finally provide a clear pathologic diagnosis by biopsy. Surgical treatment can effectively improve the survival rate and prolong the survival time of patients. The combined application of chemotherapy, radiotherapy and other treatment methods may have a better therapeutic effect on the PPATs. It still needs continuous research and exploration of more safe and more effective treatments to improve the survival rate and prognosis of patients with PPATs.

## Acknowledgments

We are most grateful to all participants in the present study.

## Author contributions

**Formal analysis:** Xiuqing Liu, Xuhan Liu, Ruirui Li.

**Investigation:** Xiuqing Liu.

**Methodology:** Xiuqing Liu.

**Writing – original draft:** Xiuqing Liu, Xuhan Liu, Ruirui Li.

**Writing – review & editing:** Weihua Zhang.
